# microRNAs and Cardiac Cell Fate

**DOI:** 10.3390/cells3030802

**Published:** 2014-08-05

**Authors:** Chiara Piubelli, Viviana Meraviglia, Giulio Pompilio, Yuri D’Alessandra, Gualtiero I. Colombo, Alessandra Rossini

**Affiliations:** 1 Center for Biomedicine, European Academy of Bolzano/Bozen, Via Galvani 31, I-39100 Bolzano, Italy; E-Mails: chiara.piubelli@eurac.edu (C.P.); viviana.meraviglia@eurac.edu (V.M.); 2 Centro Cardiologico Monzino, IRCCS, Via Parea 4, I-20138 Milano, Italy; E-Mails: giulio.pompilio@ccfm.it (G.P.); ydalessa@ccfm.it (Y.D.); gualtiero.colombo@ccfm.it (G.I.C.)

**Keywords:** microRNA, pluripotency, cardiomyocytes, cardiac reprogramming, cardiac development

## Abstract

The role of small, non-coding microRNAs (miRNAs) has recently emerged as fundamental in the regulation of the physiology of the cardiovascular system. Several specific miRNAs were found to be expressed in embryonic, postnatal, and adult cardiac tissues. In the present review, we will provide an overview about their role in controlling the different pathways regulating cell identity and fate determination. In particular, we will focus on the involvement of miRNAs in pluripotency determination and reprogramming, and specifically on cardiac lineage commitment and cell direct transdifferentiation into cardiomyocytes. The identification of cardiac-specific miRNAs and their targets provide new promising insights into the mechanisms that regulate cardiac development, function and dysfunction. Furthermore, due to their contribution in reprogramming, they could offer new opportunities for developing safe and efficient cell-based therapies for cardiovascular disorders.

## 1. Introduction

microRNAs (miRNAs ) are a class of 19–25 nucleotide (nt) non coding RNAs, evolutionarily conserved in animals [1]. They modulate gene expression post-transcriptionally by inhibiting translation and/or inducing specific mRNA degradation. The general mechanism of miRNA action is based on base pairing of the so called ‘‘seed’’ sequence (miRNA nt 2–8) with complementary sites of the target messenger RNA, mainly located in the 3'untranslated region (3'UTR), but also in the coding region [2] or in the 5'UTR [3,4,5,6]. In some cases, pairing to the 3'portion of the miRNA can enhance the binding and compensate for a possible single mismatch in the seed region [7,8]. Additional factors, such as positions of miRNA binding sites, site accessibility, RNA secondary structure, and proximity of sites for other miRNAs and RNA binding proteins can also influence miRNA-mRNA interactions [1,5,9].

miRNAs were described for the first time as post-transcriptional regulators in 1993 [10,11]. Since then, their functional relevance in most of the processes regulating development and pathophysiology has been extensively demonstrated. In the heart, miRNAs modulate key complex gene regulatory pathways involved in cardiovascular development, function and disease [5,12,13]. Regulation of miRNA expression thus represents a fundamental cellular mechanism for the control of the translation of several proteins, involved in cardiac myogenesis, morphogenesis and contractility [5,14,15]. Improving knowledge about the role of miRNAs may help finding new mechanisms and markers or targets for cardiovascular diseases.

Moreover, considering their function in controlling gene expression in cell fate determination, cellular proliferation and differentiation [16], miRNAs might represent suitable tools to modulate* ex vivo* the commitment of endogenous stem cells to specific lineage, in order to develop safe and efficient cell therapies for cardiovascular disorders.

The present review provides an overview on the role of microRNAs in cell pluripotency determination and reprogramming and specifically on cardiomyocyte cell identity and fate commitment during cardiac differentiation. 

## 2. Biogenesis and Function of miRNAs

miRNAs can be transcribed either individually or in clusters. Based on their locations in the genome, miRNAs are classified as intergenic, taking their own promoters, or exonic and intronic, which can be either co-expressed with their host genes or transcribed in the opposite orientation of the host genes, having their own cis-regulatory elements. The biogenesis of miRNAs starts with the RNA polymerase II-mediated synthesis of a precursor molecule, called primary miRNA transcript (pri-miRNA), which can encode single or multiple miRNAs [17,18]. Several miRNAs are transcribed in polycistronic units that give rise to multiple miRNAs. Some miRNAs can also be encoded by two alleles or by nearly identical genes; in this case, the related miRNAs are indicated as miR-xx-1 and miR-xx-2 [1,16]. Pri-miRNAs are processed by the endonuclease Drosha and its cofactor, DiGeorge syndrome critical region 8 (DGCR8) and converted into 60–100 nt hairpins, known as precursor miRNAs (pre-miRNAs) with imperfect base pairing stems [19,20,21]. An alternative nuclear pathway for miRNA biogenesis was also described [22] (Berezikov *et al.*, 2007): “mirtrons” are precursors spliced directly out of introns and debranched into pre-miRNA, by-passing the Drosha-DGCR8 step. The pre-miRNAs are then translocated from the nucleus to the cytoplasm by exportin 5 [23]. Once in the cytoplasm, they are further processed by the ribonuclease III enzyme Dicer, to produce imperfect miRNA duplexes (about 22 nt in length) [24]. One strand of the miRNA duplex is subsequently incorporated into a miRNA-induced silencing complex (miRISC) and becomes a mature functional miRNA, whereas the other strand can be rapidly degraded or processed to become a mature miRNA itself [25]. In the latter case, names like miR-xx-5p (from the 5' arm of the hairpin precursor) and miR-xx-3p (from the 3' arm) are used [26]. Finally, the miRNA-RISC complex recognizes specific targets and induces posttranscriptional gene silencing [27,28]. Different mechanisms have been proposed for this gene expression modulation: miRNAs can inhibit translational initiation or elongation, mark target mRNAs for degradation by deadenylation, or sequester targets into cytoplasmic P bodies [29].

More than 1,500 human miRNA genes have been identified so far and it is estimated that miRNAs regulate over 60% of the mammalian protein coding transcripts [30,31]. miRNAs exert their activity mainly through a subtle regulation of numerous targets, rather than acting with dramatic repression/activation of single genes, thus representing a sort of “fine-tuner” of the gene expression regulation. The complexity of miRNA-dependent gene regulatory circuits can be explained considering different key properties such as: each miRNA can regulate the expression of several hundred genes; individual 3' UTRs can have binding sites for multiple miRNAs, allowing cooperation between miRNAs; and miRNAs themselves can be modulated by feedback mechanisms promoted by the protein products of mRNA targets [5].

## 3. MicroRNAs in Pluripotency and Reprogramming Control

**Figure 1:**
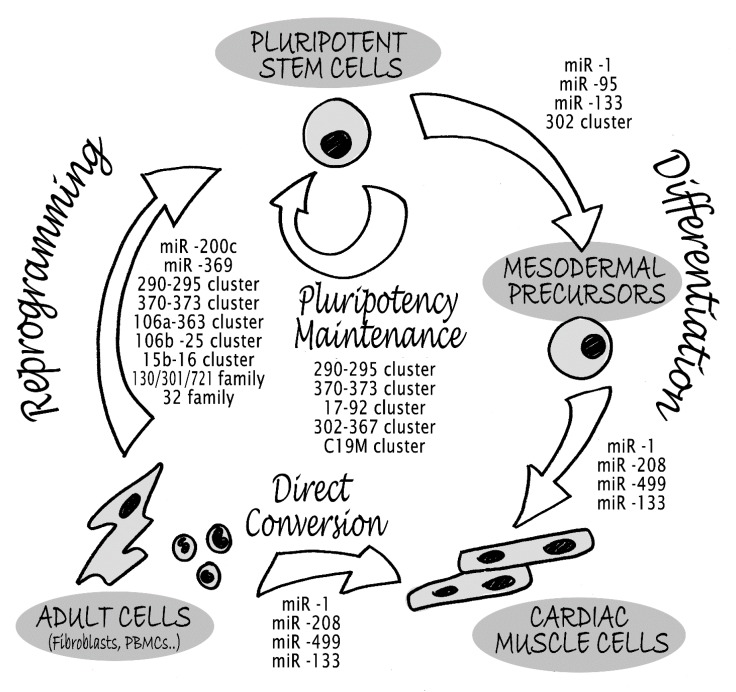
miRNAs regulating pluripotency and cardiomyogenic differentiation processes. The figure represents the different groups of miRNAs that have been shown to be involved in the following processes: reprogramming from adult cells into pluripotent stem cells, maintenance of pluripotent state, differentiation into cardiac muscle cells, through the mesodermal precursors, and direct transdifferentiation from adult cells into cardiac muscle cells.

Pluripotent stem cells are highly enriched with distinct subsets of miRNAs [32], such as miR-290-295, miR-370-373 (the orthologs to the mouse miR-290-295 cluster), miR-17-92 and miR-302–367 clusters [31,33] (Figure 1).

Their key role in the regulation of pluripotency has emerged following the evidence that pluripotent embryonic stem cells (ESC) lacking the miRNA processing machinery fail in proliferation and differentiation processes [34,35]. Moreover, Marson* et al.* demonstrated that pluripotency transcription factors (TFs), such as Octamer-binding transcription factor 4 (Oct4), Sex-determining region Y (SRY)-Box2 (Sox2) and Nanog homeobox, that are abundant in ESC, promote the expression of several miRNA clusters [36]. Among these, the miR-290-295 cluster is one of the most highly expressed in ESC [35] and represents the majority of all miRNA species in undifferentiated ESC [16]. Several members of the miR-290-295 cluster (e.g. miR-291, -294, and -295), and of the miR-302-367 cluster belong to the so-called ESC-specific cell cycle regulating (ESCC) miRNAs [30]. ESCC miRNAs contribute to the maintenance of the unique ESC cell cycle, accelerating the G1-S transition, by targeting multiple inhibitors of the Cyclin E-Cdk2 pathway that are key regulators of the cell cycle [37,38]. 

The miR-17-92 family and the miR-370-373 cluster, which share similar or identical seed sequences with ESCC miRNAs, are also associated with pluripotency and cell proliferation [39,40].

Some specie-specific miRNAs were also found in undifferentiated human ESC (hESC), such as the chromosome 19 miRNA cluster (C19MC) [41,42]. This large cluster of highly related miRNAs includes members of the miR-506, miR-515, and miR-520 families, and is expressed at low level in hESC, as observed in miRNA expression profiling studies by microarrays [41,42]. In particular, the miR-520 family seems to be involved in cell proliferation and chromatin remodeling [42]. These microarray studies also observed that several other miRNAs, not commonly described as hESC enriched, appeared to change their expression rapidly in response to the loss of pluripotency. Several of these miRNAs, such as miR-301, -101, -141,-148a, and -374, as well as many members of C19MC were lowly or moderately expressed in undifferentiated hESC [41]. Further studies are needed to determine the role of these miRNAs in hESC.

Epiblast stem cells (EpiSCs) are pluripotent cell lines derived from the postimplantation epiblast, which is the most proximal pluripotent tissue to the early somatic and germ cell precursors [43,44,45]. EpiSCs can also be obtained from primed ESCs* in vitro* (Guo* et al.*, 2009), so they represent a more advanced embryonic developmental stage in respect to the naïve ESC. Like ESCs, EpiSCs express the core pluripotency genes Oct4, Nanog, and Sox2, and are able to form teratocarcinomas and differentiate into multiple lineages* in vitro*. Mouse EpiSCs are of particular interest because human ESCs share many of their typical properties [46]. 

Despite these functional similarities, ESCs and EpiSCs differ in several distinct characteristics (for reviews, refer to [46,47,48]; in particular, they show different gene expression and miRNA profiles. miRNA expression profile revealed that several clusters associated with pluripotency were differentially expressed in mouse EpiSCs compared to mouse ESC, including miR-290-295, miR-17-92, miR-302-367, and a large repetitive cluster on chromosome 2 [49]. Specifically, EpiSCs seem to express miRNAs mapping in the miR-302-367 cluster more abundantly, while the members of the miR-290-295 and miR-17-92 clusters were found to be more highly expressed in ESCs, thus reflecting specific roles of ESCC miRNAs in the fine-tuning of pluripotency during development [49,50]. Interestingly miRNAs from the conserved miR-302-367 cluster represent the most abundant class of miRNAs in hESCs, in accordance with the proposal that hESCs could correspond to the primed rather than the naive state of pluripotency [49,51]. In addition, some other miRNAs were reported to be involved in the control of the transition from ESC to the epiblast stage, like miR-125 [52] and miR-200 family [53] that seem to be involved in the regulation of stemness maintenance and differentiation through the action of Bone morphogenic protein 4 (BMP4) and Nodal/Activin signaling pathways.

In the light of the evidence that ESCC miRNAs are highly expressed in ESC and are absent in differentiated cells, several studies evaluated the role of miRNAs in cellular reprogramming, in order to increase the induction efficiency or directly generate induced pluripotent stem cells (iPSC) from adult differentiated cells. iPSC were first generated from adult mouse and human fibroblasts in the laboratory of Shinya Yamanaka. In a landmark work, they obtained iPSC from somatic differentiated cells by transfecting a combination of a few reprogramming TFs (Oct4, Sox2, Kruppel-like factor 4 (Klf4), and c-Myc) [54], thus circumventing ethical and immunological problems associated with ESC handling. Although the mechanisms that allow the reprogramming from a differentiated to a pluripotent state are not completely clarified, it is known that the processes supporting a self-renewal program involve a reorganization of different cellular functions, including a rearrangement of microRNA expression profile.

Several studies focused on the role of miRNAs in cell fate reprogramming. In particular, Judson* et al.* first reported that the overexpression of miR-291-3p, miR-294 and miR-295, in combination with the reprogramming factors Oct4, Sox2, and Klf4, improves iPSC generation from mouse embryonic fibroblasts [55]. Enhancement in reprogramming efficiency was also obtained either with members of the miR-302 family in mouse fibroblasts [55] or with the overexpression of both the miR-302-367 cluster and miR-372 in human fibroblasts in combination with Oct3/4, Sox2, Klf4 and c-Myc [56].

In addition, other clusters were found to be involved in cellular reprogramming besides the ESCC miRNAs, like the miR-106b-25 and miR-106a-363 clusters, the miR-130/301/721 family, the miR-15b-16 cluster and the miR-32 family [31,33].

Indeed, it has been shown that the overexpression of the miR-106a-363 and miR-302-367 clusters in the presence of three reprogramming factors (Oct3/4, Sox2, and Klf4) led to an increase in efficiency of iPS cell generation from mouse fibroblasts by accelerating the mesenchymal-to-epithelial transition [57]. Notably, a recent miRNA library screening identified the miR-130/301/721 family as an important regulator of iPSC induction in the presence of Oct3/4, Sox2, and Klf4, by targeting the homeobox transcription factor Meox2 [58]. 

Most interestingly, recent findings showed that miRNAs alone could promote pluripotent reprogramming of mouse and human somatic cells without the requirement for usual pluripotency transcription factors [59,60]. 

Lin* et al.* demonstrated that the miR-302-367 cluster induced pluripotency in human cancer [61] and hair follicle cells [60] without exogenous additional reprogramming factors. In particular, these studies showed that overexpression of the miR-302-367 cluster induced not only the co-expression of Oct3/4, Sox2, and Nanog genes but also a global demethylation, which led to the reprogramming of both cancerous and normal cells into a pluripotent state. Anokye-Danso* et al.* reported that the expression of the miR-302-367 cluster by integrating retroviral vectors was sufficient to induce pluripotency of mouse and human fibroblasts and the reprogramming efficiency was two magnitudes higher compared to the usual approach using Oct4, Klf4, Sox2, and c-Myc. This study also demonstrated that valproic acid (VPA) was able to reprogram mouse fibroblasts by specifically degrading Hdac2 protein. Interestingly, VPA did not affect the reprogramming of human fibroblasts, probably due to the much lower levels of Hdac2 protein expressed in human than in mouse embryonic fibroblasts [62]. In addition, it has been reported that mouse and human adipose stromal cells may be reprogrammed to pluripotency by transient transfection of mature double-stranded miRNAs, using a combination of miR-200c plus miR-302-367 and miR-369 miRNA families [59]. This study concluded that all these three miRNAs were essential to generate iPSC from somatic cells. Indeed, miR-200c was shown to be necessary for an efficient reprogramming, due to its ability to repress the epithelial-mesenchymal transition by inhibiting TGF-β signaling [63]. Notwithstanding the foregoing, the miRNAs-based reprogramming strategy, without the addition of others pluripotency factors, is still controversial and was only reported in the above cited studies [33]. In addition, the results are often not comparable, because the reprogramming efficiency could depend on different combinations and the required optimal combination is specific according to the cell type. Further work is needed to optimize reprogramming using miRNAs alone in absence of the normally used transcription factors [64].

Some miRNA families (e.g. let-7, miR-34, miR-21 and miR-29a) interfere with reprogramming [65,66]. Inhibition of these miRNAs is associated with an enhanced reprogramming efficiency [33]. For example, knocking down multiple let-7 family members improved the iPSC generation [67].

## 4. miRNAs in Cardiomyocyte Lineage Commitment and Identity 

Developmental identity is regulated by integrated gene regulatory networks, in which several factors are involved, like for instance specific sets of TFs, epigenomic regulators, signaling mediators, and post-transcriptional regulators, including miRNAs.

There is increasing evidence that miRNAs play a crucial role in the cell fate specification during differentiation of pluripotent cells and more restricted progenitor cells. Specification can be promoted either by inhibiting specific differentiation processes or by inducing the commitment of a particular cell lineage. 

In the heart, miRNAs play key roles in the emergence and maintenance of the differentiated state of cardiomyocytes (CM) (Figure 1) and seem to be critically involved also in cardiac pathophysiology [25,68]. Expression profiling of CM derived from human ESC highlighted significant changes in several microRNAs. Among these, miR-1, miR-133, miR-208, and miR-499 were reported to be modulated during cardiac differentiation [69,70,71].

Both miR-1 and miR-133 are highly expressed in cardiac and skeletal muscle cells and are critical regulators of muscle proliferation and differentiation [72]. They seem to synergistically promote mesodermal differentiation in ESC, while suppressing differentiation into the endodermal and neuroectodermal lineage [69]. In the mammalian genome, miR-1 and miR-133a are encoded by two distinct gene clusters (miR-1-1/miR-133a-2 and miR-1-2/miR133a-1), localized in two different chromosomes. A third related cluster, encoding miR-206/mir-133b, is expressed exclusively in skeletal muscle [13,73].

miR-1 and miR-133a seem to exert opposite functions during proliferation and differentiation of muscle precursor cells. They are transcribed together in a tissue-specific manner during development and show distinct roles in modulating skeletal muscle proliferation and differentiation in cultured myoblasts* in vitro* and in *Xenopus laevis* embryos* in vivo* [72]. Chen *et al.* reported that miR-1 promotes myogenesis by targeting histone deacetylase 4 (HDAC4), a transcriptional repressor of muscle gene expression. By contrast, miR-133 enhances myoblast proliferation by repressing serum response factor (SRF) [72]. Cardiac- and skeletal-muscle-specific expression of the miR-1/133a cluster is regulated by upstream and intragenic enhancers controlled by SRF and Myocyte Enhancer Factor 2 (MEF2), two MADS-box transcription factors, as well as by the Myoblast Determination protein (MyoD) [74,75]. These transcription factors also activate protein-coding genes involved in muscle function (e.g. sarcomeric protein), thus evidencing the importance of the mutual support between miRNA and mRNA regulatory networks [5]. 

*In vitro*, miR-1 acts as a suppressor of endoderm and ectoderm differentiation and its overexpression promotes CM differentiation in mouse and human ESC, as indexed by an increase in the expression of Nkx2.5, an early cardiac marker [69]. Overexpression of miR-1 in human fibroblasts shifted their gene expression pattern toward that of muscle cells [76], accelerated the occurrence of beating areas and increased cardiac marker genes in adult cardiac progenitor cells [71]. Likewise, the overexpression of miR-1 in human iPSC gave rise to troponin T positive cells, increased the expression of the mesoderm marker mesoderm posterior 1 homolog (MESP1), and enhanced either early stage cardiac transcription factors (Nkx2.5, Islet1 (ISL1) and GATA binding protein 4 (GATA4)) and late stage sarcomeric genes (cardiac troponin T (CTNT), myosin heavy chain alpha and beta genes (Myh6 and Myh7, respectively)) during differentiation [77]. Enrichment in miR-1 was found in human CM but not in smooth muscle or in endothelial cells derived from ESC-derived multipotent cardiovascular progenitors (MCPs). miR-1 was demonstrated to exert its fate-switching action in promoting CM differentiation by targeting both frizzled family receptor 7 (FZD7) and fibroblast growth factor receptor substrate 2 (FRS2), implying its key role in inducing cardiovascular commitment from MCPs by simultaneously suppressing WNT and fibroblast growth factor (FGF) pathways [77]. Other direct targets of miR-1 involved in the regulation of cardiac differentiation were histone deacetylase 4 (HDAC4), Notch ligand Delta-like (Dll-1), and heart- and neural crest derivatives-expressed protein 2 (Hand2) transcription factor. All together, these data support a role for miR-1 in promoting sarcomerogenesis and myogenic differentiation, while simultaneously repressing the smooth muscle program.

miR-133a seems to play an opposite role compared to miR-1 in the differentiation of muscle lineage, as it inhibits the differentiation into cardiac muscle [69], but its specific role is debated. Some studies reported that overexpression of miR-133a in mouse and human ESC repressed cardiac markers [69,78]. miR-133a was found to be highly expressed in differentiated myoblasts [72], but its function in these cells is controversial. Some studies reported an enhancement of myoblast proliferation by miR-133a, targeting and repressing SRF, cyclin D2, myogenin, and Myosin Heavy Chain (MHC) [72,79]. In contrast, a suppression of myoblast proliferation by miR-133a targeting SP1 has also been reported [80]. Injection of a miR-133 inhibitor into zebrafish embryos caused a disruption of actin organization in the sarcomere [81]. 

*In vivo* studies reported that single deletion of miR-133a-1 or miR-133a-2 genes yielded phenotypically normal mice. However, deletion of both miR-133a genes caused a fetal heart phenotype with variable penetrance characterized by ventricular septum defects (VSD), increased CM proliferation, apoptosis, cardiac fibrosis, sarcomere disarray and aberrant overexpression of smooth muscle genes in the heart, probably due to the inappropriate expression of SRF and cyclin D2 [79]. Gain-of-function studies in which miR-133a was overexpressed in embryonic heart, under the control of the β-MHC promoter, further confirmed the ability of miR-133a to regulate CM proliferation: miR-133a overexpression led to VSDs, ventricular thinning, and decrease in CM proliferation [79]. Intriguingly, it has been recently demonstrated that cardiac stromal cells [82,83] isolated from right auricles acquire several properties of electrically competent cells (*i.e.* presence of a small inward sodium current and of a pacemaker current), when treated with a specific combination of epigenetic modulators. This was associated with an extensive remodeling of CStC miRNA expression profile. Of note, miR-133a was significantly up-regulated by the treatment, in agreement with the observation that miR-133a is expressed at higher levels in adult CM with respect to cardiovascular precursors [84].

It may thus be concluded that miR-133a-1 and miR-133a-2 redundantly regulate the gene expression programs required for normal cardiac growth and function and are critical for the regulation of cell proliferation and sarcomere gene expression. 

A family of intronic miRNAs, the so-called “myomiRs” is encoded by three muscle-specific myosin genes: miR-499 is encoded by the intron 20 of the Myh7B gene and shares many predicted targets with miR-208a, which in turn is encoded by the intron 28 of Myh6 and with miR-208b, which is localized into Myh7. While miR-208a is expressed only in the heart, both miR-208b and miR-499 are expressed in skeletal muscle too. Among their functions, these myomiRs control myofiber identity, muscle myosin content and muscle performance [5].

In particular, miR-499 is constitutively expressed in the heart [85] and is enhanced in cardiac committed adult cardiac progenitor cells [71] and human ESC [70]. It is involved in stress-adaptation of the adult heart [68]. Overexpression of miR-499 in human cardiac stem cells reduced the proliferation of human cardiac progenitor cells, accelerated the formation of beating embryoid bodies [71], and resulted in an enhanced myocyte differentiation when injected* in vivo* into the border zone of infarcted rat hearts [84]. In addition, inhibition of miR-499 in mouse and human ESC culture blocked cardiac differentiation [71], thus demonstrating that miR-499 is essential in the control of cardiac commitment [70,86]. miR-499 was hardly detectable in c-kit-positive cardiac stem cells, while it was highly expressed in CM [84] together with miR-1 and miR-133a. This distinctive distribution may indicate its importance in the specialization process of cardiomyocytes. As demonstrated by Hosoda* et al.*, genes involved in differentiation, such as the SRY-box containing gene 6 (SOX6) and the protein tyrosine phosphatase, non-receptor type 3 (PTBP3), are targets of miR-499 [84]. By overexpressing miR-499 in cardiac stem cells, the amount of SOX6 and PTBP3 proteins decreased. This modification corresponded to an augmented differentiation of the cells into myocyte lineage, as indicated by the expression of myocyte-specific markers Nkx2.5 and GATA4 [84]. Interestingly, miR-499 was demonstrated to be translocated through the gap junctions from mature CM to cardiac stem cells to favor the differentiation into newly formed CM [87]. This new modality of cell-to-cell communication, different from paracrine, was termed “mircrine” [87] and seems to be implicated in the control of cell fate.

Evidence of an involvement of the miR-302-367 cluster, miR-93 and miR-145 in early mesodermal differentiation has been reported [88]. The miR-302 family is expressed exclusively in early embryogenesis in vertebrates. Loss- and gain-of-function studies on hESC showed that the miR-302-367 cluster specifically induced expression of both mesodermal (Brachyury and forkhead box F1, FoxF1) and endodermal (Mix1 homeobox-like 1, Mixl1, and Sox17a) markers, thus repressing neuroectodermal differentiation and maintaining the pluripotency markers Oct4 and Nanog [89].

Foshay and Gallicano reported that, among the miR-17-92 family, miR-93 could promote early mesodermal differentiation, since its inhibitors delayed the expression of the Brachyury mesodermal differentiation marker in mouse ESC, while miR-93 mimics increased the expression of the same marker. These data were consistent with the observation of a strong miR-93 expression in mesodermal layer of mouse gastrulating embryos [90].

miR-145 was also reported to have a role in the induction of hESC differentiation into mesoderm and ectoderm lineages [91]. The overexpression of miR-145 in hESC by using a lentiviral vector resulted in an increase of the mesoderm-specific markers α-smooth muscle actin (SMA), Mixl1, NODAL and the ectoderm-specific markers β-III tubulin (TUJ1) and orthodenticle homeobox 2 (OTX2) [91]. 

## 5. Direct Myocardial Reprogramming with miRNAs

Direct conversion or transdifferentiation is the direct transformation of one differentiated cell type into another bypassing the pluripotent stage [92]. miRNAs, as potent regulators of cell fate reprogramming, have gained great interest in promoting direct cell-to-cell phenotypic conversion between differentiated cells. Jayawardena and colleagues provided the first evidence that miRNAs alone could induce the conversion of mouse cardiac fibroblasts into cardiomyocytes, both* in vitro* and* in vivo* [93]. They demonstrated that transient co-transfection of four specific cardiac miRNAs (miR-1, -133a, -208a, and -499) directed the phenotypic switch from fibroblasts to cardiomyocyte-like cells (Figure 1). Interestingly, miR-1 alone was sufficient to induce cardiac reprogramming but its effect was strongly enhanced in combination with miR-133a, -208a, and -499. Although the combination did not increase reprogramming efficiency, the four miRNAs together induced CM-like cells maturation. Importantly, treatment with JAK inhibitor I in combination with the four miRNAs enhanced reprogramming by 10 fold [94]. CM-like cells obtained after direct reprogramming* in vitro* not only expressed mature cardiac markers 3–6 days after transfection, but also displayed functional properties such as evidence of sarcomere-like structures, spontaneous calcium transients, and mechanical contraction (2–4 weeks after miRNA transfection). More importantly, cardiac reprogramming was also induced* in vivo*, by injecting lentivirus encoding the four miRNAs directly into ischemic myocardial area of transgenic mice with genetically-traced fibroblasts. Four weeks after lentiviral vector injection, evidence of direct low-pace conversion of cardiac fibroblasts into cardiomyocytes* in situ* was reported. However,* in vivo* experiments showed some limitations, since only 1% of resident cardiac fibroblasts were reprogrammed into cardiomyocyte-like cells and were detected in the scar and peri-infarct areas of the heart [93]. Nevertheless, this miRNA-based reprogramming strategy worked efficiently for mouse cardiac fibroblasts, but not for human dermal fibroblasts, which were only partially reprogrammed into cardiomyocyte-like cells. Despite a significant upregulation of cardiomyocyte markers, the reprogrammed human cardiomyocytes rarely displayed functional characteristics (e.g calcium transients), indicating that dermal induced cardiomyocyte-like cells were less mature than those obtained from cardiac fibroblasts. 

Nam* et al.* have demonstrated that human neonatal foreskin fibroblasts and adult cardiac and dermal fibroblasts could transdifferentiate into CM-like cells by forcing the expression of an optimal combination of cardiac transcription factors (GATA4, HAND2, T-box 5 (TBX5) and Myocardin) and two muscle-specific microRNAs, miR-1 and -133a [95]. This specific combination of factors and miRNAs effective for human fibroblasts was different from the defined optimal combination for cardiac reprogramming of mouse fibroblasts, which included Gata4, Hand2, Mef2c and Tbx5 [96]. Indeed, the conversion of human fibroblasts to CM required the addition of Myocardin and miR-1 and -133a, not only improving myocardial reprogramming, but also eliminating the requirement of MEF2C [79]. Two weeks after retroviral infection, reprogrammed human fibroblasts expressed multiple cardiac markers and suppressed non-myocyte genes, such as COL1A2, COL3A1, and S100A4. Notably, cardiomyocyte-like cells maintained in culture for 4–11 weeks exhibited sarcomeric organization and spontaneous calcium oscillations, and a small subset of induced cardiomyocytes derived from human cardiac fibroblasts displayed spontaneous contractions. 

In conclusion, these findings provided the proof-of-concept that miRNAs alone or in combination with cardiac specific transcription factors and/or cardiac-promoting small molecules possess the ability to induce direct conversion of fibroblasts toward cardiogenic lineage. These results have important implications in view of possible therapeutic applications for cardiac tissue regeneration. Further improvements in these miRNA-mediated strategies will lead to derive transdifferentiated cardiomyocytes for drug screening, disease modeling, and cell-replacement therapies.

## 6. miRNAs in Cardiac Development

Loss-of-function mutations of the miRNA-processing enzymes Dicer and Drosha/DGCR8 produced evidence for an essential role of miRNAs during cardiac development. Conditional deletion of Dicer using Cre recombinase, under the control of cardiomyogenic regulatory DNA sequences, resulted in lethality at different developmental stages, according to the temporal expression of the Cre transgene. For example, early deletion of Dicer using Nkx2.5 regulatory region, led to embryonic lethality due to cardiac failure at embryonic day 12.5 (E12.5) [97]. The ventricular myocardium of Nkx2.5-Cre/Dicer(fl/fl) mice was poorly developed and the heart exhibited signs of pericardial edema. This notwithstanding, several initial cardiac differentiation markers were normally expressed, like Tbx5, Hand1, Hand2, and myosin light chain 2 (Mlc2v). miRNAs are also essential for post-natal heart function, as demonstrated by Dicer deletion under the control of α-MHC promoter in cardiomyocytes. In this case, mutant mice died between post-natal day 0 and 4 (P0–P4) [98], showing dilated cardiomyopathy, heart failure, and a decreased expression level of mature miRNAs as early as E14.5. The cell number or CM size at P0 was not affected; a depressed cardiac contractility was reported, accompanied by misexpression of cardiac contractile proteins and severe sarcomere disarray [98].

In addition to Dicer deletion mutants, further studies implying deletion of DGCR8 gene supported the hypothesis of a critical role of miRNAs during neonatal heart development. Disruption of Dgcr8 gene in mice was obtained near birth using a muscle creatine kinase-promoter-driven Cre recombinase (MCK-Cre), with cardiac transgene activity declining by P10 [99]. As for Dicer mutant hearts, disruption of Dgcr8 in neonatal cardiomyocytes resulted in depressed cardiac contractility and conduction abnormalities with progression to dilated cardiomyopathy and premature death occurring within 2 months after birth [99].

Dicer mutant cardiomyocytes were also obtained at different post-natal ages, using a tamoxifen-inducible α-MHC-Cre line. Dicer deletion in young hearts (3-week-old mice) resulted in mild ventricular remodeling, severe atrial enlargement, re-activation of fetal cardiac genes and sudden cardiac death within 1 week after the start of tamoxifen treatment [100]. Dicer deletion in adult hearts (8-week-old mice) produced dramatic ventricular hypertrophy, myofibrillar disarray, cardiac fibrosis, and marked induction of fetal cardiac genes [100]. Further recent studies reported pathological ventricular remodeling and contractile dysfunction following tamoxifen-inducible Dicer deletion in 6-week-old adult mice [101].

Taken together, these studies demonstrated that miRNA biogenesis is not only crucial for the preservation of normal cardiac function and animal survival during neonatal life, but also in juvenile and adult stages for cardiac growth and maintenance of cardiac function.

miR-1 was the first to be described as implicated in heart development and it is expressed in cardiac and skeletal muscle precursors [74,97]. As stated above, miR-1 is encoded by two nearly identical genes: miR-1-1 and miR-1-2. Targeted deletion of miR-1-2 gene (with a reduction of about the 50% cardiac miR-1 expression) resulted in several cardiac defects, including defective morphogenesis, electrophysiological abnormalities, and dysregulated cell-cycle control in CM [97]. Specifically, defects in the propagation of cardiac electrical activity were observed in targeted deletion of miR-1-2 mice. It has been demonstrated that miR-1 regulates cardiac conduction by directly targeting Iroquois-Class Homeodomain Protein (IRX-5), a transcription factor that controls cardiac repolarization by the repression of Potassium Voltage-Gated Channel Subfamily D Member 2 (KCND2) [97]. Also, it has been recently shown that SRF/miR-1 feedback regulatory axis finely controls the expression of sodium/calcium exchanger-1 (NCX1), involved in regulating excitation-contraction coupling and cardiac action potential [102]. Indeed, it was demonstrated that SRF activity regulates NCX1 protein level in cardiomyocytes through direct transcriptional activation and also indirectly by controlling miR-1 expression, that targets and represses NCX1 mRNA [103]. 

Complete loss of the miR-1 resulted in lethality before weaning due to cardiac dysfunction, since miR-1-1 and miR-1-2 double knockout mice present severe myocardial sarcomeric defects, with gene expression features more similar to the smooth muscle [104]. Overexpression of miR-1 in the developing heart leads to a decreased pool of proliferating ventricular cardiomyocytes in mice, resulting in developmental arrest at embryonic day 13.5 [75]. In line with this, studies in *Drosophila* deletion mutants demonstrated a role for miR-1 in modulating cardiogenesis and muscle differentiation [105].

Other key miRNAs involved in cardiac development, are miR-133, the myomiR family, and the miR-17-92 cluster [13]. The miR-17-92 cluster generates a pri-miRNA transcript encoding six miRNAs that can be grouped into four classes, based on their seed sequences. Loss-of-function studies have highlighted critical roles for the miR-17-92 cluster during heart development in mice [106]. miR-17-92 homozygous deletion mutants died perinatally and exhibited cardiac defects like VSDs, lung hypoplasia and B-cell development defects. On the contrary, loss of the paralogous miR-106a-93 and miR-106b-25 clusters did not affect the viability of these mutants, and animals did not present cardiac dysfunctions. Nevertheless, mutants lacking both miR-17-92 and miR-106b-25 died at mid gestation with an increased severity of cardiac developmental defects, including VSDs, atrial septal defects (ASD) and thin-walled ventricular myocardium [106]. Exploring the mechanism by which the miR-17-92 cluster regulates survival of early B cell progenitors, some studies suggested that members of this cluster repress the expression of pro-apoptotic genes. Indeed, several pro-apoptotic proteins were validated as miR-17-92 cluster targets, including the phosphatase and tensin homolog (PTEN), the Transcription factor E2F1 [107,108] and the B-cell lymphoma 2 (Bcl-2) interacting mediator of cell death (Bim) [106]. In addition, gain-of-function studies in mice overexpressing miR-17 revealed a further function for the miR-17-92 cluster in the regulation of organ size, as transgenic mice displayed growth retardation in multiple organs, including the heart [109]. In this case, repression of fibronectin was proposed as a putative mechanism of action for the growth suppression mediated by miR-17 overexpression. In contrast, transgenic overexpression of the entire miR-17-92 cluster in developing cardiac cells induces cardiomyocyte hypertrophy and hyperplasia [110]. These conflicting results may be due to non-overlapping functions of different miRNAs within the miR-17-92 cluster. 

Recent expression profiling studies have revealed an upregulation of multiple members of the miR-15 family during neonatal life, [111], which coincides with CM cell cycle arrest and binucleation. The miR-15 family consists of six members, which are clustered on three separate chromosomes (miR-15a and miR-16-1, miR-15b and miR-16-2, miR-195 and miR-497). Gain- and loss-of-function studies in the mouse pointed out that the miR-15 family is involved in the induction of cardiomyocyte mitotic arrest during the neonatal period. Overexpression of miR-195* in vivo*, under the control of the β-MHC promoter, inhibited cardiomyocyte proliferation in the embryonic heart and was associated with VSDs and ventricular hypoplasia: mice developed a slow-onset dilated cardiomyopathy and died between 5 and 18 months of age [111]. In addition, there was a reduction in the number of mitotic cardiomyocytes, an increase in the number of multinucleated CM, and repression of mitotic genes [92]. Similarly, overexpression of miR-15 family members* in vitro* inhibited cardiomyocyte proliferation [111,112]. These results suggested that post-natal upregulation of the miR-15 family has an important modulating role on the cardiac cell-cycle machinery. In fact, some of the genes identified as targets of the miR-15 family are involved in cell proliferation, like checkpoint kinase 1 (Check1). In addition, miR-15 protected CM from cell death* in vitro* via regulation of Bcl-2 and sirtuin 1 (Sirt1) [113,114].

Like their host genes, miR-208a is highly expressed in mouse adult heart, whereas miR-208b is abundant in embryonic but at a low level in adult heart in physiological conditions [85,115,116]. In response to a cardiac stress such as pressure overload, β-MHC/miR-208b is upregulated in the adult heart [85,115]. Studies on miR-208a null mice reported defects in cardiac conduction. miR-208a is likely to control cardiac conduction by regulating expression levels of the gap junction protein connexin 40, the Homeodomain-only protein (Hop) and GATA4 transcription factors [115]. In line with these findings, mice overexpressing miR-208a developed cardiac hypertrophy and arrhythmias [115].

Some studies in zebrafish reported an involvement of miR-138 in cardiac maturation and patterning. Knockdown of miR-138 zebrafish embryos resulted in pericardial edema, indicative of cardiac dysfunction, impaired cardiomyocyte maturation and cardiac looping defects [117]. Cardiac patterning was also affected, given that genes normally restricted to the atrioventricular canal region resulted in being ectopically expressed in the ventricular chamber. 

Conversely, overexpression of miR-218 during early stages of zebrafish embryogenesis caused cardiac defects including incomplete looping, as well as impaired ventricular and atrial morphogenesis [118]. In addition, overexpression of this miRNA disrupted cardiac patterning and was associated with ectopic expression of the normally endothelial-restricted gene Tie2 in the atria and ventricles. MiR-138 is highly conserved from zebrafish to humans and miR-218 appears to be a vertebrate specific miRNA. Further studies are required to evaluate the role of these miRNAs in the development and morphogenesis of the mammalian four-chambered heart. 

## 7. Concluding Remarks

miRNAs are part of the gene regulatory networks that control pluripotency and proliferation as well as differentiation processes into distinct lineages. Stimulated by specific extracellular cues, signaling cascades guide the determination of cellular fates, by the activation of lineage specific transcription factors that promote the necessary cellular programs for the correct differentiation. 

In this review, we have discussed the central role of miRNAs in controlling proliferation, self-renewal and reprogramming capacity of stem cells, as well as differentiation towards cardiomyocyte lineage in cardiac differentiation commitment. 

The examined studies highlighted the crucial role for specific miRNAs in the maintenance of pluripotency of stem cells, since they are implicated in the repression of transcription factors promoting cellular differentiation. Once the commitment* versus* cardiac differentiation has started, lineage-specific miRNAs are upregulated inhibiting transcription factors specific for the self-renewal state. Furthermore, in these processes miRNAs provide an additional mechanism of regulation of specific transcriptional programs by suppressing factors that exceed a certain threshold in the differentiation and development processes.

Although the comprehensive understanding of the functions of mirRNAs is far from being completed and additional work is needed to clarify miRNA influence in regulating cell identity and fate determination, identification of cardiac-specific miRNAs and their targets provide new promising insights into the mechanisms that regulate cardiac development, function and dysfunction. In addition, considering miRNAs role in control reprogramming, they could represent suitable tools for developing safe and efficient cell-based therapies for cardiovascular disorders. 
